# Postnatal growth of preterm infants during the first two years of life: catch-up growth accompanied by risk of overweight

**DOI:** 10.1186/s13052-021-01019-2

**Published:** 2021-03-16

**Authors:** Junyan Han, Yuan Jiang, Jun Huang, Yue Zhang, Ying Zhang, Yi Zhang, Xiaotian Chen, Yun Li, Weili Yan

**Affiliations:** 1 Children’s Hospital of Fudan University, 399 Wanyuan Road, Minhang District, Shanghai, 201102 China; 2 National Children’s Medical Center, 399 Wanyuan Road, Minhang District, Shanghai, 201102 China; 3 Shanghai Minhang District Maternal and Child Health Care Hospital, 805 Gudai Road, Minhang District, Shanghai, 201102 China

**Keywords:** Preterm, Growth, Catch-up growth, Overweight

## Abstract

**Background:**

Early postanal growth of preterm infants has many effects on early and late health. However, evidence on growth pattern in Chinese preterm infant population during early life is insufficient. This study aims to describe the growth trajectory, catch-up growth, and risk of overweight of preterm infants during the first 2 years of life in a Chinese community population.

**Methods:**

All preterm infants (*n* = 10,624) received routine childcare in one primary maternal and child healthcare network in 8 years were included. Body weight and length/height at corrected age (CA) 40 weeks, CA 3 months, 6 months, 9 months, 12 months, 18 months, and 24 months were extracted and converted to z-scores based on the World Health Organization (WHO) standards. According to the intrauterine growth status, infants were divided into small for gestational age (SGA), appropriate for gestational age (AGA), and large for gestational age (LGA) infants. Changes of z-score were used to describe the growth velocity. Generalized estimating equation (GEE) model was used to analyze growth trajectory trends over time.

**Results:**

Body weight and length/height were overall above the WHO standards during the first 2 years of life. Z-score increased significantly by 0.08 (*95% CI*: 0.06–0.10) in weight and 0.07 (*95% CI*: 0.04–0.09) in length/height from CA 40 weeks to 3 months and then levelled off until CA 24 months after adjustment. Almost 90% of AGA and LGA infants achieved growth targets (≥25th percentile of WHO standards), and over 85% of SGA infants achieved catch-up growth (≥10th percentile of WHO standards) before CA 24 months. However, the risk of overweight appeared during this period, with the proportion of infants with the risk of overweight being at the peak at CA 3 months (25.6% of all preterm infants and 39.4% of LGA infants). Growth trajectories of SGA showed increasing trends, but those of LGA showed decreasing trends during the first 2 years.

**Conclusions:**

Body weight and length/height of preterm infants are above the WHO standards in the Chinese community population during the first 2 years of life. Catch-up growth is accompanied by risk of overweight as early as CA 3 months.

(349 words)

**Supplementary Information:**

The online version contains supplementary material available at 10.1186/s13052-021-01019-2.

## Background

More than one in 10 of the world’s babies born in 2014 were preterm infants, resulting in an estimated 14.8 million preterm births [1]. Because of the vast population base, the number of preterm infants in China ranked second in the world [1, 2]. Although the survival rate of preterm has increased over past decades along with the development of perinatal and neonatal medicine, they are still more susceptible to growth restriction, delayed motor, and language development in later life [3, 4].

Growth trajectory monitoring is an essential part of children’s healthcare, especially for preterm infants. Previous studies on growth trajectory have shown that preterm and/ or low birth weight infants were shorter and lighter than term infants of the same postmenstrual age (PMA). Meanwhile, they often have a rapid growth or catch-up growth period after post-discharge [5, 6]. Early catch-up growth is beneficial for neurodevelopment [7]. Belfort et al.’s study in 945 preterm infants showed rapid growth from term age to 4 months was associated with better intelligence quotient at 18 years old [8]. However, associations between excessive growth in infancy and overweight/obesity, insulin resistance, and elevated blood pressure in childhood or adulthood have been reported in recent studies [9, 10]. A study in Korean population reported a phenomenon of early accelerated growth or “early catch-up growth” in preterm infants, which might be associated with Asian cultural values and childrearing practices [11]. Nevertheless, evidence on growth pattern or growth trajectory in Chinese preterm infants is still limited.

This study aims to describe the growth trajectory, catch-up growth, and risk of overweight of preterm infants during the first 2 years of life in a community-based Chinese population, and to compare the growth differences between small for gestational age (SGA) infants, appropriate for gestational age (AGA) infants, and large for gestational age (LGA) infants.

## Methods

### Data source and subjects

Study data were extracted from a routine database of the Maternal and Child Healthcare Network of Minhang district in Shanghai, China. It was a population-based database launched by the government, which recorded all infants’ routine healthcare information living in this district constituted of 13 communities for 2.53 million permanent residents. This study included all preterm infants (born before 37 weeks of gestation) who received routine healthcare during the period from January 1, 2010, to December 31, 2017. Infants with a missed or ambiguous birth date, birth weight, or without growth parameter data (body weight and length/height) were excluded. Corrected age (CA) was used in all analyses, defined as the additional age from 40 weeks of PMA. Follow-up data were grouped into CA 40 weeks (40 weeks of PMA), 3 months, 6 months, 9 months, 12 months, 18 months, and 24 months based on their original date of visits. The institutional scientific research department approved this analysis protocol. Personal identification information was removed from the extracted dataset for analyses.

### Measurements and definitions

Body weight and length/height were measured and recorded by trained pediatricians and nurses in community healthcare centers at each routine visit. An electronic scale with 0.01 kg accuracy was used to measure body weight. Length/height was measured using a length board in a supine position, or a measurement system in standing position reading to the nearest 0.1 cm. For each infant, z-score of measurements were computed using the Lambda-Mu-Sigma (LMS) parameters based on the international standards [12, 13]. The Fenton preterm growth standard (from 22 to 50 weeks of gestation) [12] was used to calculated z-scores at birth, and the World Health Organization (WHO) standards for children 0–5 years old [13] were used to calculated z-scores from CA 40 weeks to 24 months. According to the intrauterine growth status, all preterm infants were classified as SGA, AGA, and LGA infants, defined as birth weight < 10th percentile, 10th–90th percentile, and > 90th percentile on the Fenton Growth Chart, respectively [12].

In this study, we defined that growth target was achieved for AGA and LGA infants when the weight or length/height for age ≥ 25th percentile of the WHO standards. The growth target for SGA infants was weight or length/height for age ≥ 10th percentile of the WHO standards, which meant achieving catch-up growth [13, 14]. Infants whose weight for length > 90th percentile of the WHO standard were defined as at risk of overweight [13, 14].

### Statistical analyses

Measurement values were included in analyses only if the original date of visits were within 2 weeks around the specific follow-up points. Those measurement values beyond mean ± three standard deviations (SD) were removed as outliers. Demographics and clinical characteristics were described as mean and SD for continuous variables and absolute numbers with percentages for categorical variables. Growth measurements of our population and the WHO standards were compared using *t*-test for each follow-up time point. Generalized estimating equation (GEE) models were used to analyze trajectory trends of these growth measurements over time. Growth velocity was defined as the change of z-score over the periods [15, 16] using the lincom function of GEE models. Gestational age, gender, birth weight z-score, and intrauterine growth status was adjusted as covariates. Stata 16.0 software (Stata Corp, College Station, TX, USA) was used for all the statistical analyses, and the significance level was set at 5% (two-tailed). Figures were drawn using GraphPad Prism 8.0 for Windows (GraphPad Software, La Jolla California, USA).

## Results

### Clinical characteristics

A total of 10,705 preterm infants met the inclusion criteria. Among them, 80 infants without any follow-up measurement and one infant missing the birth information were excluded. Finally, 10,624 infants were included in the analyses, with gestational age 35.0 ± 1.8 weeks (rang 24^+ 5^–36^+ 6^ weeks) and birth weight 2463 ± 520 g. Basic characteristics were shown in Table 1. Among all preterm infants, the majority were late preterm infants (> 34 weeks of gestation, 82.7%) and AGA infants (86.0%). Over 75% of infants had highly educated parents (one of the parents received an education in college or above). The body weight z-score was 0.04 ± 0.91 at birth.

**Table 1:** Characteristics of all preterm infants [mean ± SD or n(%)]

Characteristics	Total (*n* = 10,624)
Male, n (%)	5895 (55.5)
Primiparity, n (%)	5955 (56.1)
Gestational age, weeks	35.0 ± 1.8
Birth weight, g	2463 ± 520
Birth weight z-score^a^	0.04 ± 0.91
Caesarean section, n (%)	6376 (60.0)
Maternal age, years	29.7 ± 4.9
Paternal age, years	31.7 ± 5.9
≥ college (maternal education)	6945 (76.6)
≥ college (paternal education)	7102 (78.3)
Subgroup by gestational age	
< 28 weeks, n (%)	40 (0.4)
28 ~ 31^+ 6^ weeks, n (%)	642 (6.0)
32 ~ 33^+ 6^ weeks, n (%)	1161 (10.9)
34 ~ 36^+ 6^ weeks, n (%)	8781 (82.7)
Subgroup by birth weight	
< 1000 g, n (%)	35 (0.3)
1000-1499 g, n (%)	437 (4.1)
1500 ~ 2499 g, n (%)	4810 (45.3)
> 2500 g, n (%)	5342 (50.3)
Intrauterine growth status	
SGA, n (%)	681 (6.4)
AGA, n (%)	9141 (86.0)
LGA, n (%)	802 (7.6)

^a^ Z-scores were computed according to the Fenton Preterm Infants Growth Charts;

*SD* standard deviation; *SGA* small for gestational age; *AGA* appropriate for gestational age; *LGA* large for gestational age

### Growth of preterm infants from CA 40 weeks to CA 24 months

**Fig. 1:**
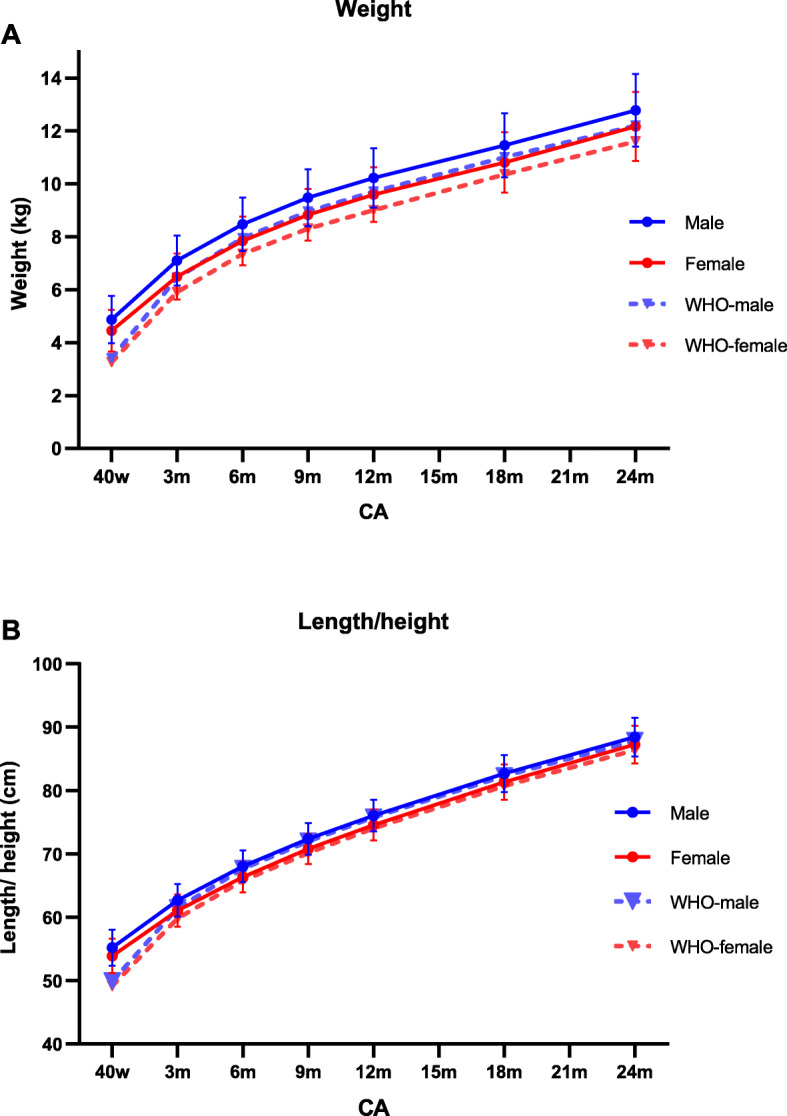
Growth trajectories of preterm infants from CA 40 weeks to 24 months. **a** Growth trajectory of body weight in preterm infants and the mean level of the WHO standard. **b** Growth trajectory of length/height in preterm infants and the mean level of the WHO standards. CA, corrected age; WHO, World Health Organization

As shown in Fig. 1 and Additional file 1, body weight increased 7.90 kg in males and 7.72 kg in females from CA 40 weeks to CA 24 months. The mean increment in length/height was 33.2 cm and 33.4 cm for males and females, respectively. All growth parameters in our study population were significantly above the WHO standards, especially in weight (all *P*-values < 0.001). Growth appears to be faster in male preterm infants than female during the first 2 years of life.

Table 2 showed an estimated change of z-scores (growth velocities) during the six time periods after adjustment. From CA 40 weeks to 3 months, z-score of body weight and length/height increased significantly by 0.08 [*95% confidence interval (CI)*: 0.06–0.10] and 0.07 (*95%CI*: 0.04–0.09), respectively (all *P*-values < 0.001). After that, z-score of body weight declined significantly among periods from CA 3 to 18 months (all *P*-values < 0.001), while with an exception for the change from CA 18 to 24 months (*P* = 0.071). For length/height, the growth velocity also showed a declining trend from CA 6 to 18 months (all *P*-values < 0.001), while remaining stable from CA 3 to 6 months and from CA 18 to 24 months (*P* > 0.05). Some covariates, including gestational age, birth weight, gender, and intrauterine growth status, showed significant contributions in growth (Additional file 2).

**Table 2:** Change of z-scores in preterm infants from CA 40 weeks to 24 months

Corrected age	Z-score change of weight		Z-score change of length/height	
	*Difference (95% CI)*^a^	*P*-value	*Difference (95% CI)*^a^	*P*-value
Overall	*–*	< 0.001	*–*	< 0.001
CA 40 weeks-3 months	0.08 (0.06–0.10)	< 0.001	0.07 (0.04–0.09)	< 0.001
CA 3–6 months	− 0.07 (− 0.09- -0.05)	< 0.001	0.01 (− 0.01–0.04)	0.310
CA 6–9 months	− 0.10 (− 0.12- -0.08)	< 0.001	− 0.07 (− 0.10- -0.05)	< 0.001
CA 9–12 months	− 0.08 (− 0.10- -0.06)	< 0.001	− 0.09 (− 0.12- -0.07)	< 0.001
CA 12–18 months	− 0.17 (− 0.20- -0.15)	< 0.001	− 0.11 (− 0.14- -0.08)	< 0.001
CA 18–24 months	− 0.02 (− 0.06–0.00)	0.071	0.00 (− 0.03–0.04)	0.918

^a^ Gestational age, gender, birth weight z-score, and intrauterine growth status were adjusted;

*CA* corrected age; *CI* confidence interval

### Achieving growth target and risk of overweight

**Fig. 2:**
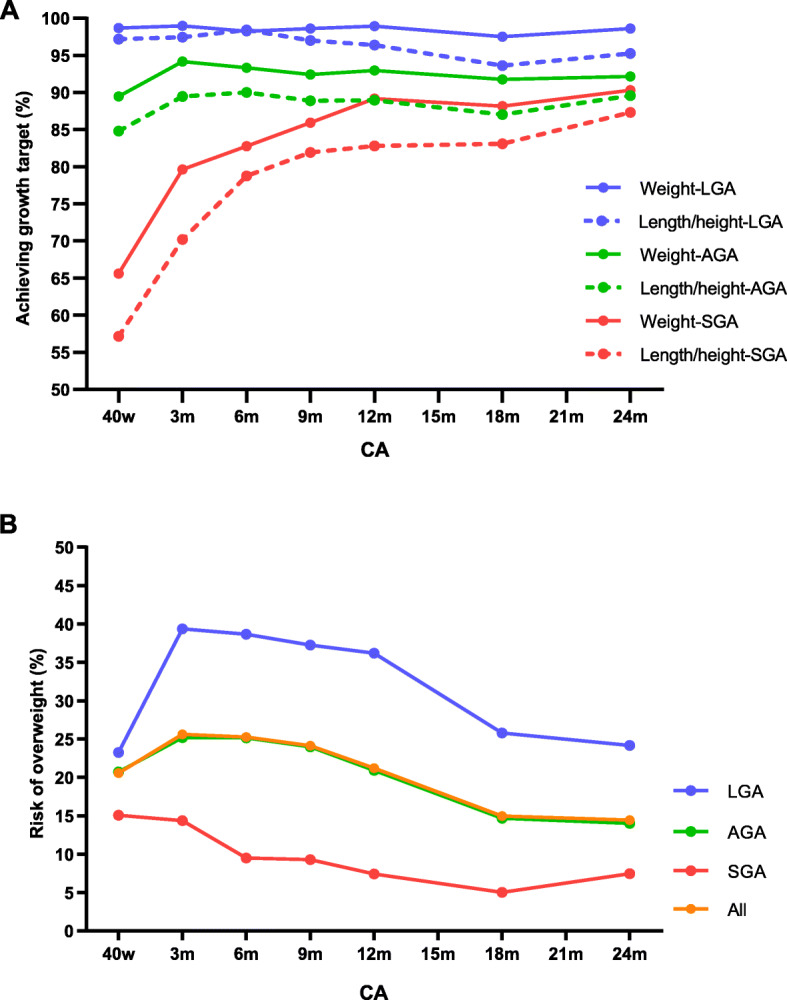
Achieving target growth and risk of overweight from CA 40 weeks to 24 months in preterm infants. **a** The proportion of infants who achieved target growth or achieved catch-up growth. **b** The proportion of infants with risk of overweight. CA, corrected age; SGA, small for gestational age; AGA, appropriate for gestational age; LGA, large for gestational age

At CA 24 months, 92.2 and 89.6% of AGA infants, and 98.6 and 95.3% of LGA infants achieved growth target in weight and length/height, respectively (Fig. 2a), and most of them occurred before CA 3 months. Afterward, the proportion of infants achieving growth target kept stable. As for SGA infants, a significant trend of catch-up growth was observed across the 2 years after birth. Especially for the first year of life, the proportion of infants who achieved catch-up growth (reach 10th percentile of the WHO standards) increased from 65.6 to 89.2% in weight and from 57.2 to 82.8% in length/height. By the CA 24 months, most SGA infants achieved catch-up growth (90.3% in weight and 87.3% in length/height).

The risk of overweight appeared accompanied by catch-up growth (Fig. 2b). At CA 40 weeks, 20.6% of infants were at risk of overweight. This proportion increased to the peak (25.6%) at CA 3 months, and then gradually decreased to 14.5% at CA 24 months. Risk of overweight even occurred in SGA infants, and the proportion ranged from 5.0 to 15.1% during the first 2 years of life. The proportion of infants with the risk of overweight was significantly higher in LGA infants than that in AGA and SGA infants. Especially at CA 3 months, up to 39.4% of LGA infants were at risk of overweight.

### Growth of SGA, AGA, and LGA preterm infants

**Fig. 3:**
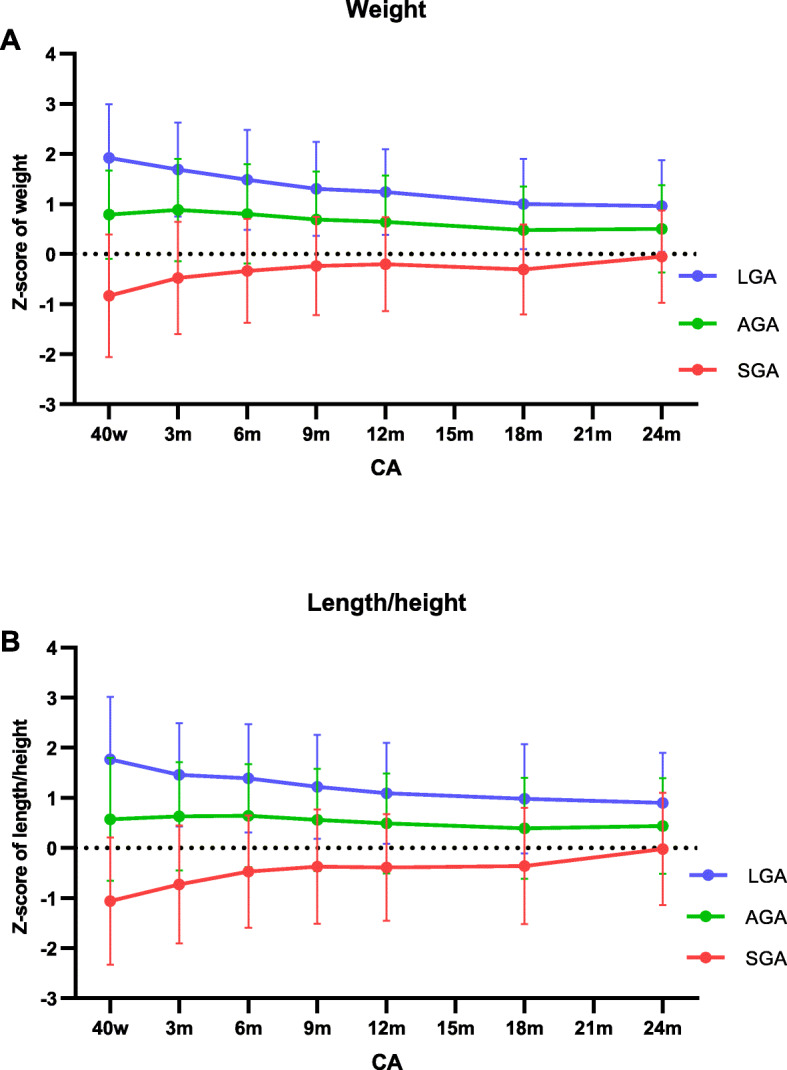
Growth trajectories of LGA, AGA, and SGA preterm infants from CA 40 weeks to 24 months. **a** Growth of body weight in LGA, AGA, and SGA infants. **b** Growth of length/height in LGA, AGA, and SGA infants. CA, corrected age; SGA, small for gestational age; AGA, appropriate for gestational age; LGA, large for gestational age

Figure 3 showed z-scores of body weight and length/height in SGA, AGA, and LGA infants. Differences between these subgroups gradually decreased during the first 2 years of life.

For SGA infants, body weight and length/height z-scores were significantly lower than the WHO standards by 0.83 ± 1.23 and 1.06 ± 1.27 at CA 40 weeks. From CA 40 weeks to CA 24 months, these z-scores showed overall increasing trends, with the mean of z-score increasing by 0.78 in weight and 1.04 in length/height. When CA 24 months, these measurements (body weight z-score = − 0.05 and length/height z-score = − 0.02, Additional file 3) were closed to the mean level of WHO standard (z-score = 0). The period with the fastest growth velocity was from CA 40 weeks to 3 months, weight z-score increased by 0.37 (*95%CI*: 0.28–0.45, *P*-value< 0.001) and length/height z-score increased by 0.37 (*95%CI*: 0.16–0.49, *P*-value < 0.001) (Table 3).

**Table 3:** Growth velocities of SGA, AGA, and LGA preterm infants from CA 40 weeks to 24 months

Subgroup	Corrected age	Weight		Length/height	
		*Difference (95% CI)*^a^	*P*-value	*Difference (95% CI)*^a^	*P*-value
SGA	Overall	–	< 0.001	–	< 0.001
	CA 40 weeks- 3 months	0.37 (0.28–0.45)	< 0.001	0.37 (0.16–0.49)	< 0.001
	CA 3–6 months	0.14 (0.06–0.23)	0.001	0.27 (0.15–0.38)	< 0.001
	CA 6–9 months	0.05 (−0.03–0.15)	0.243	0.07 (−0.06–0.19)	0.278
	CA 9–12 months	0.04 (− 0.06–0.13)	0.433	− 0.00 (− 0.13–0.13)	0.992
	CA 12–18 months	−0.10 (− 0.20–0.00)	0.057	0.03 (− 0.10–0.17)	0.640
	CA 18–24 months	0.15 (0.01–0.29)	0.035	0.25 (0.07–0.44)	0.008
AGA	Overall	–	< 0.001	–	< 0.001
	CA 40 weeks- 3 months	0.08 (0.07–0.11)	< 0.001	0.07 (0.05–0.10)	< 0.001
	CA 3–6 months	−0.07 (− 0.09- -0.05)	< 0.001	0.00 (− 0.02–0.03)	0.751
	CA 6–9 months	−0.10 (− 0.12- -0.08)	< 0.001	−0.07 (− 0.10- -0.05)	< 0.001
	CA 9–12 months	−0.09(− 0.11- -0.07)	< 0.001	−0.10 (− 0.12- -0.07)	< 0.001
	CA 12–18 months	−0.17 (− 0.20- -0.15)	< 0.001	−0.12 (− 0.14- -0.09)	< 0.001
	CA 18–24 months	−0.03 (− 0.06- − 0.00)	0.032	-0.00 (− 0.04–0.04)	0.999
LGA	Overall	–	< 0.001	–	< 0.001
	CA 40 weeks- 3 months	− 0.22(− 0.29- -0.14)	< 0.001	− 0.29 (− 0.38- -0.20)	< 0.001
	CA 3–6 months	−0.21 (− 0.29- -0.14)	< 0.001	−0.08 (− 0.17–0.01)	0.069
	CA 6–9 months	−0.20 (− 0.29- -0.12)	< 0.001	−0.18 (− 0.27- -0.08)	< 0.001
	CA 9–12 months	−0.07 (− 0.16–0.02)	0.129	−0.13 (− 0.23- -0.03)	0.009
	CA 12–18 months	−0.24 (− 0.33- -0.15)	< 0.001	−0.13 (− 0.24- − 0.03)	0.012
	CA 18–24 months	-0.03 (− 0.15–0.09)	0.667	−0.08 (− 0.22–0.05)	0.218

^a^ Gestational age, gender, birth weight z-score, and intrauterine growth status were adjusted;

*SGA* small for gestational age; *AGA* appropriate for gestational age; *LGA* large for gestational age; *CA* corrected age; *CI* confidence interval

For AGA infants, the means of body weight and length were overall above the WHO standards from CA 40 weeks to 24 months (Fig. 3 and Additional file 3). Z-score increased significantly by 0.08 (*95%CI*:0.07–0.11) and 0.07 (*95%CI* 0.05–0.10) from CA 40 weeks to 3 months in body weight and length/height, respectively (all *P*-values< 0.001). After that, z-scores of measurements showed overall declining trends until CA 24 months (Table 3).

For LGA infants, z-score of these measurements showed overall declining trends during the first 2 years (Table 3). From CA 40 weeks to 24 months, the mean of z-scores declined by 0.96 in weight and 0.87 in length/height, respectively (Additional file 3). Z-score of body weight declined significantly from CA 40 weeks to 9 months (all *P*-values < 0.001) and from CA 12 to 18 months (decline by 0.24, *95%CI* = -0.33- -0.15, *P*-value < 0.001). Length/height z-scores also showed declining trends from CA 40 weeks to 3 months (decline by 0.29, *95%CI* = -0.38- -0.20, *P*-value < 0.001) and from CA 6 to 18 months (all *P*-values < 0.001) (Table 3).

## Discussion

This study provides evidence on the growth trajectory during the first 2 years of life in a large Chinese preterm population, showing that the body weight and length/height were overall higher than the WHO standards. Almost 90% of AGA infants and over 90% of LGA infants achieved target growth, and over 85% of SGA infants achieved catch-up growth before CA 24 months. However, over a quarter of infants had been at risk of overweight as early as CA 3 months, and this proportion reached 39.4% in LGA infants. To date, we are the first reporting early overweight risk accompanying catch-up growth in the Chinese preterm infant population.

Our study noticed that growth trajectories of preterm infants were consistently over the WHO standards from CA 40 weeks to CA 24 months, especially for weight. Recent studies in Chinese population also found that growth rate of preterm infants was higher than that in term infants during the first year of life [17, 18]. Several possible reasons might explain this phenomenon. Firstly, this study data was from a preterm infant population in community healthcare center, most infants were late preterm infants who were less likely to develop serious diseases and might growth better after birth. Secondly, we believed that rapid growth is directly related to the childrearing practice of their parents, giving their preterm babies more care and feeding to make up the low birth weight. In addition, the “One Child” policy in China before 2016 might be another explanation. Parents and caregivers devote more effort to their only child among these people. A point that the heavier the baby is, the healthier he/she will be is widely accepted [18].

Catch-up growth was important for preterm infants to reduce the risk of stunning and achieve a better neurodevelopment outcome in later life [19–21], especially to SGA infants who had a higher risk of growth retardation and delayed development during infancy and childhood [22, 23]. Olbertz et al.’s study showed that few SGA infants could achieve catch-up growth after age two [24]. Currently, no consensus has reached on which catch-up growth pattern is optimal for preterm infants [25–27]. In our study, most AGA/LGA infants achieved growth targets before the first 3 months. Nevertheless, it occurred lately in other studies, at 6 months or 11–12 months [28, 29]. The difference in later outcomes between early and later achieving growth target is unclear. Further study is needed to answer this question. In addition, we found that it took more time for SGA preterm infants to achieve catch-up growth. Early aggressive nutrition strategy might be necessary for SGA infants to timely achieve the optimal catch-up growth and healthy development, which is in line with a similar point in previous studies [23, 30].

One of our main findings was that the growth was accompanied by the risk of overweight in this Chinese preterm infant population. Over a quarter of infants in this population are at risk of overweight as early as CA 3 months, and the overweight risk is even higher in LGA preterm infants. Excessive weight gains during early life in preterm infants were linked with a higher prevalence of type 2 diabetes and cardiovascular diseases in adulthood [31–34]. Kerkhof et al. observed that gain in weight for length of preterm infants during the first 3 months after term age was associated with greater body fat percentage, waist, serum cholesterol, and low-density lipoprotein in early adulthood [35]. Therefore, given these reports of adverse effects on adult health, controlling the risk of overweight during the same period of promoting optimal catch-up growth in preterm infants is becoming a challenge in primary child healthcare practice [3, 21]. Different risk-balance considerations in different preterm infants are needed, and one size might not fit all [36].

### Strengths and limitations

Based on a local community-based child healthcare network, this study has the largest sample size of Chinese preterm infant population on growth trajectory of preterm infants compared with published reports before, while some limitations are inevitable. Firstly, the study sample consists of a greater proportion of late preterm infants (82.7%), higher than that in a published nationwide survey [37]. This may be partly explained by the fact that very preterm infants or very low birth weight infants are more likely to be admitted in the neonatal intensive care unit (NICU) of tertiary hospitals and followed up at there, rather than at community healthcare centers. Besides, the proportion of late preterm infants becomes greater (2562/2824, 90.7%) at CA 24 months. Thus, the estimations of growth status and trends in our study may be overestimated and more generalizable for late preterm infants or preterm infants born relatively healthy. In addition, data of maternal diseases history, neonatal diseases, and feeding information are not available in our study, their influences on the status and trends of growth are not investigated and warrant further prospective well-designed studies.

## Conclusions

Body weight and length/height of preterm infants in the Chinese community population are above the WHO standards during the first 2 years of life, and catch-up growth is accompanied by risk of overweight, which occurred as early as CA 3 months. Although the optimal growth pattern is not fully established, more attention is needed to promote the proper catch-up growth of preterm infants for better long-term outcomes.

## Supplementary Information

**Additional file 1.** Growth of preterm infants from CA 40 weeks to 24 months compared with the WHO standards (mean ± SD).

**Additional file 2.** Covariates associated with growth velocity of preterm infants.

**Additional file 3.** Body weight and length/height z-scores of SGA, AGA, and LGA infants from CA 40 weeks to 24 months (mean ± SD).

## Data Availability

The datasets used and/or analyzed during the current study are available from the corresponding author on reasonable request.

## Abbreviations

AGA: Appropriate for gestational age
CA: Corrected age
CI: Confidence interval
GEE: Generalized estimating equation
LGA: Large for gestational age
LMS: Lambda-Mu-Sigma
NICU: Neonatal intensive care unit
PMA: Postmenstrual age
SD: Standard deviation
SGA: Small for gestational age infants
WHO: World Health Organization
