# Supplementary material for: Postnatal growth of preterm infants during the first two years of life: catch-up growth accompanied by risk of overweight
Source: Ital J Pediatr. 2021 Mar 16;47:66. doi: 10.1186/s13052-021-01019-2 (PMC7968173; doi:10.1186/s13052-021-01019-2)
Supplement: Supplementary file 1 — Additional file 1. Growth of preterm infants from CA 40 weeks to 24 months compared with the WHO standards (mean ± SD). [file 13052_2021_1019_MOESM1_ESM.pdf]

Additional file 1. Growth of preterm infants from CA 40 weeks to 24 months compared with the WHO standards (mean±SD)

Age	N	Weight (kg)				Length/height (cm)			
		Study data	WHO standard	*t*	*P*	Study data	WHO standard	*t*	*P*
**Male**									
CA 40 weeks	4661	4.87±0.89	3.40±0.50	47.85	<0.001	55.19±2.83	49.90±1.90	53.59	<0.001
CA 3 months	4835	7.10±0.95	6.45±0.75	13.64	<0.001	62.68±2.58	61.45±2.05	9.51	<0.001
CA 6 months	4130	8.47±1.01	7.95±0.85	10.08	<0.001	68.03±2.51	67.65±2.15	3.01	0.003
CA 9 months	3751	9.47±1.08	8.95±0.95	9.41	<0.001	72.36±2.50	71.95±2.25	3.20	0.001
CA 12 months	3624	10.22±1.12	9.70±1.10	8.90	<0.001	76.03±2.51	75.75±2.35	2.18	0.029
CA 18 months	3241	11.45±1.21	11.00±1.20	7.15	<0.001	82.68±2.94	82.30±2.70	2.51	0.012
CA 24 months	1583	12.77±1.38	12.20±1.40	15.81	<0.001	88.44±3.06	87.85±3.05	3.52	<0.001
**Female**									
CA 40 weeks	3741	4.45±0.79	3.25±0.45	42.45	<0.001	53.86±2.73	49.15±1.85	47.66	<0.001
CA 3 months	3873	6.49±0.87	5.90±0.70	13.82	<0.001	61.02±2.57	59.80±2.10	9.67	<0.001
CA 6 months	3394	7.84±0.92	7.35±0.85	10.65	<0.001	66.35±2.41	65.75±2.25	4.99	<0.001
CA 9 months	3007	8.83±0.98	8.30±1.00	10.63	<0.001	70.77±2.44	70.15±2.45	5.02	<0.001
CA 12 months	2862	9.59±1.04	9.00±1.10	11.09	<0.001	74.54±2.46	74.00±2.60	4.30	<0.001
CA 18 months	2613	10.81±1.15	10.35±1.25	7.71	<0.001	81.32±2.80	80.70±2.90	4.31	<0.001
CA 24 months	1241	12.17±1.30	11.60±1.40	7.80	<0.001	87.26±2.97*	86.40±3.20	5.15	<0.001

CA, corrected age; WHO, World Health Organization; SD, standard deviation.
