# Supplementary material for: Postnatal growth of preterm infants during the first two years of life: catch-up growth accompanied by risk of overweight
Source: Ital J Pediatr. 2021 Mar 16;47:66. doi: 10.1186/s13052-021-01019-2 (PMC7968173; doi:10.1186/s13052-021-01019-2)
Supplement: Supplementary file 2 — Additional file 2. Covariates associated with growth velocity of preterm infants. [file 13052_2021_1019_MOESM2_ESM.pdf]

Additional file 2. Covariates associated with growth velocity of preterm infants

Factors	Weight z-score change		Length/height z-score change	
	β (95%CI)	***P***	***β (95%CI)***	***P***
Gestational age	0.08 (0.07-0.09)	<0.001	0.09 (0.08-0.09)	<0.001
Birth weight z-score	1.96 (1.90-2.03)	<0.001	1.76 (1.69-1.83)	<0.001
Gender	-0.05 (-0.08-0.03)	<0.001	0.03 (-0.00-0.05)	0.061
Intrauterine growth status	0.28 (0.24-0.33)	<0.001	0.23 (0.19-0.29)	<0.001

CI, confidence interval.
