# Supplementary material for: Postnatal growth of preterm infants during the first two years of life: catch-up growth accompanied by risk of overweight
Source: Ital J Pediatr. 2021 Mar 16;47:66. doi: 10.1186/s13052-021-01019-2 (PMC7968173; doi:10.1186/s13052-021-01019-2)
Supplement: Supplementary file 3 — Additional file 3. Body weight and length/height z-scores of SGA, AGA, and LGA infants from CA 40 weeks to 24 months (mean ± SD). [file 13052_2021_1019_MOESM3_ESM.pdf]

Additional file 3. Body weight and length/height z-scores of SGA, AGA, and LGA infants from CA 40 weeks to 24 months (mean ± SD)

Age	Weight			Length/height		
	SGA	AGA	LGA	SGA	AGA	LGA
CA 40 weeks	-0.83±1.23	0.79±1.15	1.92±1.07	-1.06±1.27	0.57±1.23	1.77±1.25
CA 3 months	-0.48±1.12	0.88±1.02	1.69±0.94	-0.73±1.18	0.63±1.08	1.46±1.03
CA 6 months	-0.34±1.04	0.80±0.99	1.48±1.00	-0.47±1.12	0.64±1.03	1.39±1.08
CA 9 months	-0.24±0.98	0.69±0.96	1.30±0.94	-0.37±1.14	0.56±1.02	1.22±1.04
CA 12 months	-0.20±0.94	0.64±0.93	1.24±0.86	-0.39±1.06	0.49±1.00	1.09±1.01
CA 18 months	-0.31±0.90	0.48±0.87	1.00±0.90	-0.36±1.16	0.39±1.01	0.98±1.09
CA 24 months	-0.05±0.92	0.50±0.87	0.96±0.92	-0.02±1.12	0.44±0.96	0.90±1.00

CA, corrected age; SGA, small for gestational age; AGA, appropriate for gestational age; LGA, large for gestational age; SD, standard deviation.
